# The ARIQUELI study: potentiation of quetiapine in bipolar I nonresponders with lithium versus aripiprazole

**DOI:** 10.1186/1745-6215-14-190

**Published:** 2013-06-27

**Authors:** Giovani Missio, Doris Hupfeld Moreno, Fernando Fernandes, Danielle Soares Bio, Márcio Gehardt Soeiro-de-Souza, Domingos Rodrigues dos Santos, Denise Petresco David, Luis Felipe Costa, Frederico Navas Demétrio, Ricardo Alberto Moreno

**Affiliations:** 1 Department and Institute of Psychiatry, Clinicas Hospital, University of Sao Paulo School of Medicine, Mood Disorder Unit (GRUDA), Rua Dr. Ovídio Pires de Campos, 785, Third Floor,North Wing, Room 12, São Paulo, 05403-010, Brazil

**Keywords:** Bipolar, Refractory, Aripiprazole, Lithium, Potentiation, Augmentation

## Abstract

**Background:**

The treatment of bipolar disorder (BD) remains a challenge due to the complexity of the disease. Current guidelines represent an effort to assist clinicians in routine practice but have several limitations, particularly concerning long-term treatment. The ARIQUELI (efficacy and tolerability of the combination of lithium or aripiprazole in young bipolar non or partial responders to quetiapine monotherapy) study aims to evaluate two different augmentation strategies for quetiapine nonresponders or partial responders in acute and maintenance phases of BD treatment.

**Methods/Design:**

The ARIQUELI study is a single-site, parallel-group, randomized, outcome assessor-blinded trial. BD I patients according to the DSM-IV-TR, in depressive, manic/hypomanic or mixed episode, aged 18 to 40 years, are eligible. After diagnostic assessments, patients initiated treatment in phase I with quetiapine. Nonresponders or partial responders after 8 weeks are allocated into one of two groups, potentiated with either lithium (0.5 to 0.8 mEq/l) or aripiprazole (10 or 15 mg). Patients will be followed up for 8 weeks in phase I (acute treatment), 6 months in phase II (continuation treatment) and 12 months in phase III (maintenance treatment). Outcome assessors are blinded to the treatment. The primary outcome is the evaluation of changes in mean scores on the CGI-BP-M between baseline and the endpoint at the end of each study phase.

**Discussion:**

The ARIQUELI study is currently in progress, with patients undergoing acute treatment (phase I), potentiation (phase II) and maintenance (phase III). The study will be extended until January 2015. Trials comparing lithium and aripiprazole with potentiate treatment in young BD I nonresponders to quetiapine in monotherapy can provide relevant information on the safety of these drugs in clinical practice. Long-term treatment is an issue of great importance and should be evaluated further through more in-depth studies given that BD is a chronic disease.

**Trial registration:**

ClinicalTrials.gov identifier: NCT01710163

## Background

Bipolar disorder (BD) treatment is a focus of ongoing research to further understanding of the clinical features and possible pathophysiology of the disease. The multivariate factors associated with the etiology of BD, coupled with the variability of clinical presentation, hamper the determination of a specific treatment with optimized outcomes (efficacy and tolerability). Difficulties in BD treatment include delay in diagnosis, high levels of comorbidity, frequent treatment nonadherence and high risk of relapse/recurrence (particularly in the presence of residual symptoms) [1].

Most currently available treatments focus on the acute phase and use a reduction in symptoms ≥50% from baseline as a measure of response. In fact, some responders to treatment continue to experience significant subsyndromic symptoms. A small number of studies have reported remission rates, defined as ≥2 months with no significant signs or symptoms of the disorder [2]. Fewer studies, however, have reported remission during the acute phase through to the maintenance phase of treatment and its predictors [3], which are of great clinical significance. Recent data show that BD treatment under special conditions was associated with full remission in only one-half of the patients and that nearly one-half of the recovered patients relapsed at least once during a 2-year follow up [4]. Maintenance treatment is necessary in BD due to the high mortality, morbidity risk and social and professional impairment associated with poor outcomes [5].

As monotherapy, quetiapine has proven more effective than lithium in the treatment of depression at doses ranging from 150 to 600 mg [6,7], with no significant difference found between 300 and 600 mg doses [8]. The drug has also shown similar efficacy in the treatment of mania at doses of between 600 and 800 mg compared with lithium after 4 weeks and with haloperidol after 84 days [9-11], although response in mania was slower when compared with haloperidol [11]. To date, quetiapine is the only antipsychotic medication with evidence of efficacy across all phases of bipolar disorder [7], and was recently indicated as the first choice in monotherapy for the treatment of bipolar depression [12,13].

On the other hand, lithium is the first-line choice for the maintenance treatment of BD, particularly for classic (euphoric) mania and bipolar depression, according to many open controlled studies, with additional clinical effects such as antisuicidal properties, augmentation and treatment of acute unipolar depression and recurrent depression [14]. Some studies have shown lithium to be more specific for manic and less effective for depressive episodes [15] while an accumulating body of evidence tends to support its specificity in psychiatric usage, particularly in patients with classic BD.

In Brazil, lithium is the first treatment choice in all BD phases, representing a suitable and affordable treatment option. However, treatment under the Brazilian Public Health System (Sistema Único de Saúde) has financial limitations, where atypical antipsychotics and some anticonvulsants are currently not made available for use in BD. Few controlled studies on the most-used treatments for the general population are therefore conducted compared with trials involving the latter medications, which the pharmaceutical industry has an interest in promoting [16].

Aripiprazole is an atypical antipsychotic with a single mechanism of action that shows partial agonist activity at dopamine D_2_ and D_3_ and serotonin 5-HT_1A_ receptors, as well as antagonist activity at serotonin 5-HT_2A_ receptors [17,18]. The drug is US Food and Drug Administration approved for the treatment of mania and maintenance of BD 1, and has proven effective in several trials for the adjunctive treatment of bipolar depression and major depressive disorder [19-22].

Despite the drug’s proven efficacy in the treatment of manic episodes [23], results of aripiprazole monotherapy in bipolar depression have been poor, with benefits not persisting beyond 8 weeks [24]. Some data, albeit not robust, suggest the drug’s efficacy as an adjunct for potentializing treatment in bipolar I depression, although it is unclear whether this response persists beyond the acute phase [25].

Information in the general literature regarding augmentation of treatments initially refractory in trials, as well as on the combination of atypical antipsychotics in acute and maintenance phases, remains scarce.

## Methods/Design

### The ARIQUELI project

The ARIQUELI (efficacy and tolerability of the combination of lithium or aripiprazole in young bipolar non or partial responders to quetiapine monotherapy) study is a randomized trial designed to assess the add-on therapies of lithium or aripiprazole for augmentation of quetiapine treatment.

### Design of the ARIQUELI study

The two key elements of the ARIQUELI project were based on the rationale described above. For the first element, the disorder was considered as a whole; that is, patients with different clinical presentations (such as depression, mania or mixed episodes) shall receive the same treatment. The treatment focus is relapse/recurrence prevention as opposed to treating a particular phase. Patients are to undergo long-term follow-up given that BD is a chronic and recurrent disorder. The second key point is that comparing the augmentation strategy using lithium versus aripiprazole should help clarify the role of atypical antipsychotic drugs in the treatment of BD.

This is a single-site, parallel-group, randomized, outcome assessor-blinded trial. The study protocol was reviewed and approved by the appropriate institutional review board in accordance with the standards and guidelines established in the current amendment of the Declaration of Helsinki, and consistent with good clinical practice and applicable regulatory requirements. Written informed consent was obtained from all patients prior to any study-related activities. Methodological details of all phases of the ARIQUELI study have been presented in accordance with the Consolidated Standard of Reporting Trials 2010 Statement [26].

The local institutional review board approved the study (Hospital das Clinicas Ethical Committee, CAPPESQ #26436). All subjects and/or family members provided written informed consent before entry into the study.

### Interventions

After diagnostic assessments, the patients initiate treatment with quetiapine for 8 weeks as described above. For acute treatment, patients shall receive a starting dosage of 100 mg on day 1, to be increased by 100 mg/day to 300 mg on day 3. The 300 mg dose shall be subsequently adjusted until reaching the most effective dose according to clinical criteria. The patient dose shall be determined according to individual tolerability, and a maximum dose of 800 mg will be allowed for this study. Patients showing remission or >50% reduction in baseline scores on the Hamilton Depression Scale and the Young Mania Rating Scale shall proceed to the maintenance phase for a further 18 months. Patients showing score reductions <50% on the scales will be allocated to one of the arms of the augmentation phase. Group I will receive lithium at the standard dose: starting at 300 mg daily, the weekly dose will be adjusted according to blood serum level (between 0.5 and 0.8 mEq/l) according to efficacy and tolerability. Group II will receive aripiprazole: starting at 10 mg daily, the dose will be adjusted up to 15 mg daily according to efficacy and tolerability. The only concomitant medications permitted are lorazepam at doses of 0.5 to 4 mg/day and biperiden at 2 to 8 mg/day.

### Eligibility

Patients are recruited from within the Institute of Psychiatry, Clinicas Hospital of the University of Sao Paulo School of Medicine. BD I patients according to DSM-IV-TR [2], in depressive, manic/hypomanic or mixed episode, aged 18 to 40 years, are eligible for the study. To be included, patients must score at least 8 points on the Hamilton Depression Scale or 10 points on the Young Mania Rating Scale. Patients with comorbid conditions are allowed to participate due to the real clinical study approach (‘more likely naturalistic study’). Patients in previous use of quetiapine and lithium or quetiapine and aripiprazole combinations will not be included. Other exclusion criteria include presenting with schizophrenia, mental retardation (IQ <90) or unstable clinical conditions.

Eligible patients currently under pharmacological treatments have to undergo a washout period according to the medication in use: 1 week for antidepressants, antipsychotics, lithium, valproate, carbamazepine and other anticonvulsants; 2 weeks for irreversible MAOI; and 4 weeks for fluoxetine and clozapine.

### Sample size and randomization

Sample size was calculated by comparing the mean difference in Clinical Global Impression for Bipolar Disorders (CGI-BP-M) scores between baseline and endpoint for the two groups. Comparison using Student's *t* test showed that a sample of 50 patients (25 in each group) attains an 80% power (at 5% significance level) for detecting a difference of 0.8 standard deviations, considered a large effect size [27]. Owing to the exploratory nature of this study and the difficulties regarding compliance in long-term trials, this was deemed an acceptable value [27].

The random allocation sequence was computer generated by a biostatistician. Patients were enrolled by clinicians who have their code revealed by the research monitor when assigned to interventions. Blinding of outcome assessors is ensured by keeping their evaluations independent from those of the clinicians.

### Study phases

Patients will be followed up for 8 weeks in phase I (acute treatment), 8 weeks in phase II (potentiation treatment) and shall continue in phase III (maintenance) until study endpoint at 2 years. Scale raters will be blind to the treatment. Only patients that respond will continue to phase 3, with response determined according to the initial symptoms score in phase I. Patients in phase III using quetiapine alone that present a new episode of any polarity, as detected on two consecutive visits, will start phase II (potentiation treatment). Patients that show no response after phase II will be dropped from the study.

The definition of clinical course is defined as follows: no response, reduction ≤25% in severity of symptoms; partial response, improvement of 26 to 49% in symptoms; response, reduction ≥50% in severity of symptoms; remission, minimal or no symptoms for at least 1 week, Hamilton Depression Scale ≤7 and Young Mania Rating Scale ≤9; sustained remission, ≥8 weeks of remission; relapse/recurrence, return of the signs and symptoms for the syndrome as per criteria; and worsening, return of symptoms at subsyndromal level [28-30].

### Primary outcomes

Primary outcome will consist of the evaluation of changes in mean scores on the CGI-BP-M between baseline and endpoint at the end of each phase of the study.

### Secondary outcomes

Secondary outcome will include the proportion of patients that achieve remission and response to each treatment at the end of each phase of the study, according to improvement on the rating scales (Hamilton Depression Scale; Montgomery–Åsberg Depression Rating Scale; Young Mania Rating Scale) and in CGI-BP-M [31]. The CGI-BP-M is a clinician rating scale modified for BD for assessing treatment response and consists of three subscales evaluating severity of mania, depression and global disease symptoms.

Other outcome parameters include safety and tolerability, quality of life and social adjustment, and cognitive impairment. These parameters will be evaluated by comparing the endpoint measures for each phase with baseline values. Safety and tolerability were determined according to the clinical evaluation of adverse effects and score on the UKU side-effect rating scale [32]. The UKU comprises a 48-item clinician rated scale evaluating side effects in psychic, neurologic, autonomic and other domains. Quality of life and social adjustment were measured with the WHOQoL-BREF [33] and the Social Adjustment Scale [34]. These instruments were previously translated and validated into Portuguese [35,36]. The WHO-QOL-BREF is a 26-item self-report scale comprising four domains: physical, psychological, social, and environmental. The Social Adjustment Scale is a 54-question self-report instrument that measures instrumental and expressive role performance over the previous 2 weeks. Cognitive impairment was measured neuropsychological tests (Wisconsin Card Sorting Test, Stroop Color Word Test [37], Wechsler Abbreviated Scale of Intelligence [38], Trail Making Test [37] and others), for which versions are available in Portuguese. All investigators received appropriate training, and inter-rater reliability is periodically assessed.

### Planned analyses

The primary analysis will entail evaluation of mean scores on the CGI-BP-M between baseline and endpoint in both groups. In addition, differences shall be evaluated by comparing the two treatment groups according to the total number of patients in full remission at the study endpoint and the reason for and timing of drop out. Continuous data will be represented as mean and standard deviation whereas categorical variables will be described by a table of frequencies. The results of all statistical comparisons of the treatment groups will be expressed as two-sided *P* values rounded to three decimal places. The criterion for statistical significance in all comparisons will be *P* ≤0.05.

Continuous variables will be compared using repeated-measures of analysis of variance, with the treatment group and study phase as factors. Rates of response, remission and dropouts will be compared between the two groups using Pearson's chi-square test for categorical data. Dichotomous measures will also be compared using odds ratios and 95% confidence limits.

The correlation of clinical issues, quality of life, social adjustment and cognitive impairment will be evaluated by Pearson's correlation.

## Discussion

Trials comparing specific treatment efficacy in BD (head to head) can show relevant information in clinical practice.

Typical limitations in clinical trials include: specific clinical forms of BD (mania/mixed or depression) for each treatment tested; scant information available on how treatment in the acute phase should progress to maintenance or which factors in acute treatment predict recurrence during maintenance treatment; and heterogeneity of patients, considering the course of illness (chronic versus nonchronic) in clinical trials can mask important treatment implications for specific populations (younger versus older, for example).

Owing to the substantial increase in treatment options, specific guidelines and algorithms are used in an effort to enhance the cost-effectiveness of care by minimizing the number of treatment options employed [28]. Despite limitations inherent to the use of these algorithms, they represent the available data concerning the levels of evidence for each treatment option.

Further evidence-based knowledge concerning augmentation strategies and maintenance therapy using combination treatments is needed in clinical practice.

## Current trial status

The ARIQUELI study is currently in progress, with patients in phases I, II and III, and is to be extended for a further 2 years.

## Abbreviations

BD: Bipolar disorder; CGI-BP-M: Clinical Global Impression for Bipolar Disorders; DSM-IV-TR: Diagnostic and Statistical Manual of Mental Disorders, 4th Edition, Text Revision.

## Competing interests

The authors declare that they have no competing interests.

## Authors’ contributions

RAM and GM have made substantial contributions to the conception and design and have given final approval of the version to be published. DHM, FF, DSB MGS-d-S, DJRdS Jr, DPD, LFC and FND have been involved in drafting the manuscript or revising it critically for important intellectual content. All authors read and approved the final manuscript.
